# A Bacteroidetes locus dedicated to fungal 1,6-β-glucan degradation: Unique substrate conformation drives specificity of the key endo-1,6-β-glucanase

**DOI:** 10.1074/jbc.M117.787606

**Published:** 2017-05-01

**Authors:** Max J. Temple, Fiona Cuskin, Arnaud Baslé, Niall Hickey, Gaetano Speciale, Spencer J. Williams, Harry J. Gilbert, Elisabeth C. Lowe

**Affiliations:** From the ‡ Institute of Cell and Molecular Biosciences, Newcastle University, Newcastle upon Tyne NE4 2HH, United Kingdom and; the § School of Chemistry and Bio21 Molecular Science and Biotechnology Institute, University of Melbourne, Parkville, Victoria 3010, Australia

**Keywords:** carbohydrate-binding protein, glycosidase, glycoside hydrolase, microbiome, X-ray crystallography

## Abstract

Glycans are major nutrients available to the human gut microbiota. The *Bacteroides* are generalist glycan degraders, and this function is mediated largely by polysaccharide utilization loci (PULs). The genomes of several *Bacteroides* species contain a PUL, PUL_1,6-β-glucan_, that was predicted to target mixed linked plant 1,3;1,4-β-glucans. To test this hypothesis we characterized the proteins encoded by this locus in *Bacteroides thetaiotaomicron*, a member of the human gut microbiota. We show here that PUL_1,6-β-glucan_ does not orchestrate the degradation of a plant polysaccharide but targets a fungal cell wall glycan, 1,6-β-glucan, which is a growth substrate for the bacterium. The locus is up-regulated by 1,6-β-glucan and encodes two enzymes, a surface endo-1,6-β-glucanase, BT3312, and a periplasmic β-glucosidase that targets primarily 1,6-β-glucans. The non-catalytic proteins encoded by PUL_1,6-β-glucan_ target 1,6-β-glucans and comprise a surface glycan-binding protein and a SusD homologue that delivers glycans to the outer membrane transporter. We identified the central role of the endo-1,6-β-glucanase in 1,6-β-glucan depolymerization by deleting *bt3312*, which prevented the growth of *B. thetaiotaomicron* on 1,6-β-glucan. The crystal structure of BT3312 in complex with β-glucosyl-1,6-deoxynojirimycin revealed a TIM barrel catalytic domain that contains a deep substrate-binding cleft tailored to accommodate the hook-like structure adopted by 1,6-β-glucan. Specificity is driven by the complementarity of the enzyme active site cleft and the conformation of the substrate. We also noted that PUL_1,6-β-glucan_ is syntenic to many PULs from other Bacteroidetes, suggesting that utilization of yeast and fungal cell wall 1,6-β-glucans is a widespread adaptation within the human microbiota.

## Introduction

The *Bacteroides* are successful colonizers of the human gut, in large part because of their ability to rapidly adapt their metabolism to allow utilization of a wide variety of complex polysaccharides from both the diet and the host (1–4). The *Bacteroides* glycan degradation systems consist of genes arranged into co-transcribed loci called polysaccharide utilization loci (PULs).^4^ PULs are typically expressed at low levels in the absence of target glycan. However, when a substrate glycan is encountered, the corresponding PUL is rapidly up-regulated, often up to 1000-fold, driven by recognition of a specific oligosaccharide or monosaccharide cue (1). Surface enzyme(s) and glycan-binding proteins (SGBPs) orchestrate degradation of polysaccharides into smaller oligosaccharides that can be imported by the SusCD-like complex, a TonB-dependent membrane transporter (5). In the periplasm, additional enzymes depolymerize the imported oligosaccharides into their component monosaccharides, which are transported into the cytoplasm and then metabolized. The enzymes that degrade these glycans are mainly glycoside hydrolases (GHs); uronic acid-containing polysaccharides are depolymerized with the assistance of polysaccharide lyases. GHs are grouped into sequence-based families on the CAZy database (www.cazy.org) (6).^5^ Within these families the enzyme fold, catalytic apparatus, and mechanism are largely conserved. Some of the GH families have been divided into sequence-related subfamilies, which can provide insight into the sequence motifs that confer the substrate specificities evident in these related enzymes (7, 8).

Recently, in addition to plant- and host-derived glycans, carbohydrates produced by microbes have been shown to be a source of nutrients for *Bacteroides* sp., and in particular *Bacteroides thetaiotaomicron. B. thetaiotaomicron* is able to degrade the extracellular polysaccharide of *Lactobacillus* spp., and the cell wall α-mannan from fungal species such as *Saccharomyces cerevisiae* and *Candida albicans* (9, 10). The ability to use microbial sources of glycans as nutrients may confer nutritional resilience upon *B. thetaiotaomicron* and related organisms in the face of a variable supply of dietary carbohydrates. α-Mannan is an outer layer of the fungal cell wall in the species described above and covers skeletal layers of β-glucan and chitin. The heavily decorated mannoproteins of the cell wall are cross-linked through their glycosylphosphatidylinositol anchor to chains of 1,6-β-glucans that are in turn linked to both the 1,3-β-glucan and chitin chains (11).

When *B. thetaiotaomicron* is cultured on yeast extract, in addition to the up-regulation of loci that orchestrate α-mannan degradation, an additional PUL defined as PUL_1,6-β-glucan_ is activated during early exponential phase (12). This locus encodes just two enzymes, which belong to GH families 3 (GH3) and 30 subfamily 3 (GH30_3). Although 1,6-β-glucanase is the only activity reported for enzymes within GH30_3, the majority of these GHs are fungal in origin and likely to be transglucosidases involved in cell-wall remodeling. Within the fungal mycoparasite *Trichoderma harzanium*, GH30_3 enzymes contribute to the ability of the organism to antagonize plant pathogenic fungi (13, 14). One bacterial GH30_3 enzyme has been characterized, from the marine bacterium *Saccharophagus degradans*, which is active on 1,6-β-glucan as well as the branched algal polysaccharide laminarin (1,3;1,6-β-glucan) (15). Given the role of 1,6-β-glucan in the cross-linking of mannoproteins into the fungal cell wall matrix, this glucan might comprise a target for bacteria that degrade α-mannan, because cleavage of the 1,6-β-glucosidic bonds would enable release of mannoproteins. Additionally, 1,6-β-glucan is found in edible fungi such as the basidiomycetes *Agaricus bisporus* (common mushroom) and *Lentinula edodes* (Shiitake mushroom), and thus these mushrooms comprise another source of the polysaccharide for *B. thetaiotaomicron* and, more widely, the human gut microbiota.

Although 1,6-β-glucans are common components of the human diet through intake of yeast cell wall and edible fungi, little is known of how these glycans are utilized by the gut microbiota. More broadly, little is known about the enzymes that degrade 1,6-β-glucans, and there is no structural data for any GH30_3 enzyme. Here we have tested the hypothesis that PUL_1,6-β-glucan_ in *B. thetaiotaomicron* plays a role in the degradation and utilization of yeast 1,6-β-glucans and not plant 1,3;1,4-β-mixed linked glucans as previously proposed (1). Our data show that this locus orchestrates the degradation of 1,6-β-glucan, and this enables *B. thetaiotaomicron* to utilize this fungal polysaccharide. The surface-located endo-1,6-β-glucanase is shown to be critical for growth of the bacterium on 1,6-β-glucan. The crystal structure of the endo-1,6-β-glucanase shows that substrate recognition is mediated by shape complementarity of the substrate-binding cleft and the hooked U-shaped conformation of 1,6-β-glucan, rather than through extensive hydrogen-bonding interactions with the polysaccharide.

## Results and discussion

### PUL_1,6-β-glucan_ orchestrates the degradation and utilization of 1,6-β-glucan by B. thetaiotaomicron

**Figure 1.**
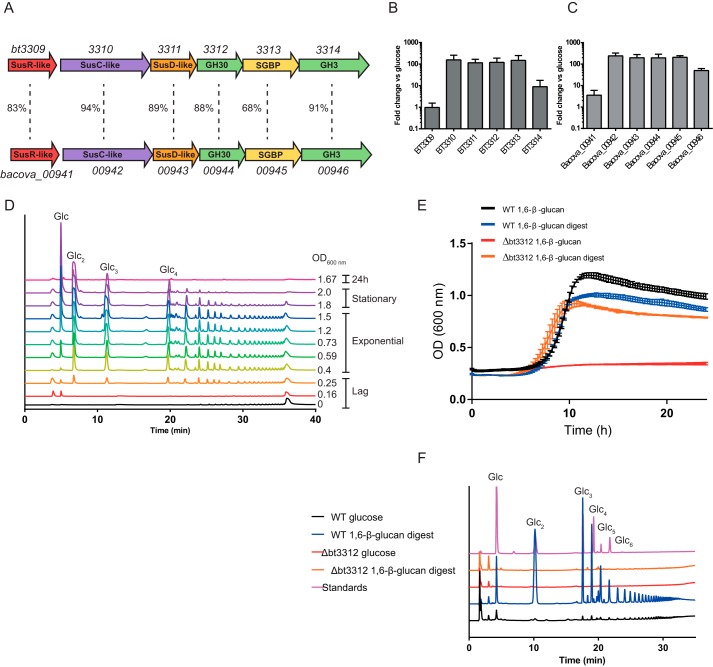
**The 1,6-β-glucan polysaccharide utilization loci from *B. thetaiotaomicron* and *B. ovatus*.** *A*, schematic of the 1,6-β-glucan polysaccharide utilization loci (PUL_1,6-β-glucan_). The percentage identity at protein level between the PULs is indicated. *B* and *C*, RT-PCR of PUL_1,6-β-glucan_ genes in *B. thetaiotaomicron* (*B*) and *B. ovatus* (*C*) grown on 5 mg ml^−1^ glucose or 1,6-β-glucan, shown as fold change in expression *versus* glucose. *D*, HPAEC-PAD analysis of soluble glycans in the culture supernatant during growth of *B. thetaiotaomicron* on 1,6-β-glucan as shown in *E*. The *A*_600_ at the time of sample is indicated. Oligosaccharides were eluted from the column with a 20% gradient of 100 mm NaOH. *OD*, optical density. *E*, growth of *B. thetaiotaomicron* WT or *bt3312* deletion strain (Δ*bt3312*) on minimal medium plus 0.5% (w/v) 1,6-β-glucan, or 1,6-β-glucan digested with 50 nm BT3312 for 5 min and then boiled to inactivate enzymes. *F*, HPAEC-PAD analysis of whole cell assays of WT or Δ*bt3312* glucose grown cells (*black* and *red*) and WT or Δ*bt3312* 1,6-β-glucan digest grown cells (*blue* and *orange*) against 2 mg ml^−1^ 1,6-β-glucan. Oligosaccharides were eluted from the column with a 60% gradient of 100 mm NaOH.

When *B. thetaiotaomicron* was cultured on the complex tryptone-yeast extract-glucose (TYG) medium, a suite of PULs were up-regulated compared with glucose minimal medium, including the locus PUL_1,6-β-glucan_ (10, 12). PUL_1,6-β-glucan_ was predicted to extend from *bt3309* to *bt3314* (Fig. 1*A*) and is predicted to encode five proteins: a SusR type regulator (BT3309), a SusCD-like pair (BT3310–11), and enzymes belonging to GH30_3 (BT3312) and GH3 (BT3314). BT3311, BT3312, and BT3313 each possess a type II signal peptide with a canonical lipoprotein box and are thus likely located on the surface of the bacterium. Because BT3313 contains multiple DUF5016 domains (also referred to as immunoglobulin domains), it is predicted to function as a SGBP (Fig. 1*A*) (12). The yeast-derived glycans in TYG medium include 1,6-β-glucan (also known as pustulan), 1,3-β-glucan, α-mannan, and chitin. *B. thetaiotaomicron* is unable to grow on laminarin, whereas the α-mannan-degrading apparatus is encoded by three PULs that are distinct from PUL_1,6-β-glucan_ (10), suggesting that the locus may target pustulan. To test this hypothesis *B. thetaiotaomicron* was cultured on pustulan, and transcription of the five genes in this locus were evaluated by RT-PCR. The gene encoding the PUL regulator BT3309 was not activated by pustulan; *bt3314* was up-regulated 10-fold; and transcription of the other genes in the locus increased ∼100-fold. The PUL was not activated when *B. thetaiotaomicron* was cultured on α-mannan. These data indicate that PUL_1,6-β-glucan_ encodes the pustulan degrading apparatus of *B. thetaiotaomicron*. This assumption was confirmed when the bacterium was shown to be unable to grow on pustulan when the gene encoding the predicted surface GH30_3 enzyme (*bt3312*) was deleted from the chromosome (Fig. 1*E*).

### Contribution of surface proteins to 1,6-β-glucan degradation

#### 

##### BT3312 is a endo-1,6-β-glucanase

GH family 30 contains enzymes that are active on a variety of β-linked glycans, including xylan, glucuronoxylan, glucosylceramide, and 1,6-β-glucan. The fungal cell wall contains 1,6-β-glucan, consistent with the location of BT3312 within GH30_3, a subfamily which, to date, is comprised exclusively of 1,6-β-glucanases. When cultured on 1,6-β-glucan, *B. thetaiotaomicron* released glucooligosaccharides into the supernatant during exponential growth, which correlates with the predicted endo-1,6-β-glucanase activity of the surface enzyme BT3312 (Fig. 1, *D* and *E*).

**Figure 2.**
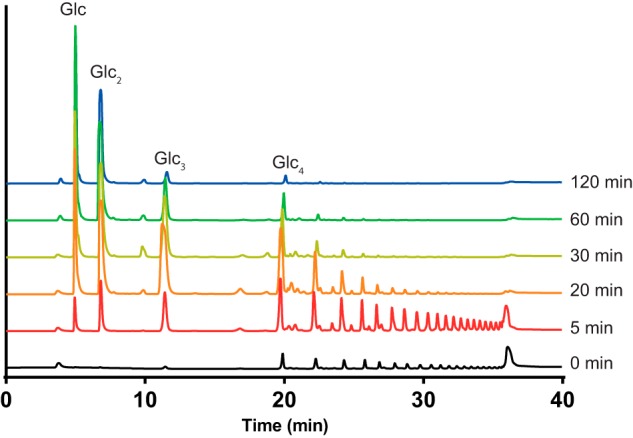
**HPAEC-PAD analysis of BT3312.** BT3312 (50 nm) was incubated with 2 mg ml^−1^ 1,6-β-glucan at 37 °C as described under “Materials and methods.” Samples were taken at the indicated time points and analyzed by HPAEC-PAD.

The catalytic activity of BT3312 was tested against a range of polysaccharides. The enzyme showed no activity on 1,3-β-glucan, 1,4-β-glucan, or 1,6-β-galactan but was active on the 1,6-β-glucan pustulan, producing a range of oligosaccharides in the initial phases of the degradative process (Fig. 2). Thus, BT3312 is a canonical and highly specific endo-1,6-β-glucanase. The limit products generated by BT3312 were primarily glucose and gentiobiose (1,6-β-glucobiose), suggesting the enzyme is active on oligosaccharides as small as glucotriose (Fig. 2). Substrate depletion assays with 1,6-β-glucooligosaccharides showed activity on oligosaccharides with a degree of polymerization ranging from 3 to 8 (Table 1). The moderate 4-fold increase in activity from glucotriose to glucooctaose suggests that the enzyme contains only three major subsites. This view is consistent with the observation that 1,6-β-glucotetraose was initially hydrolyzed to glucotriose, gentiobiose, and glucose in a molar ratio of 1:2:1 (data not shown), which showed that the substrate could bind productively in the two possible binding modes that encompass −2, −1, and +1 (*i.e*. −3 to +1 and −2 to +2; the scissile glycosidic bond links the sugars at −1 and +1) (16). Thus, −3 and +2 are not essential for substrate binding, indicating that the only functionally significant subsites in the enzyme substrate-binding cleft extend from −2 to +1. The inability of BT3312 to hydrolyze gentiobiose, which would require binding at the −1 and +1 subsites for cleavage to occur, reveals the important contribution the −2 subsite makes to productive substrate recognition, typical of endo-acting retaining glycanases (17).

**Table 1:** **Kinetic parameters for wild type and variants of BT3312**

	Substrate	*k*_cat_/*K_m_**^a^*	Relative activity
**Enzyme variant**			
Wild type	1,6-β-Glucan (pustulan)	1776 ± 20.6	1
Wild type	1,3-β-Glucan	0	0
Wild type	1,4-β-Glucan	0	0
Wild type	1,6-β-Galactan	0	0
E238A	1,6-β-Glucan (pustulan)	0	0
H281A	1,6-β-Glucan (pustulan)	10 ± 1	0.006
N282A	1,6-β-Glucan (pustulan)	17 ± 1	0.009
D286A	1,6-β-Glucan (pustulan)	475 ± 30	0.27
E339A	1,6-β-Glucan (pustulan)	0	0
E339Q	1,6-β-Glucan (pustulan)	0	0
W345A	1,6-β-Glucan (pustulan)	7 ± 1	0.004
C393S	1,6-β-Glucan (pustulan)	9 ± 1	0.005
C396S	1,6-β-Glucan (pustulan)	55 ± 3	0.03

**Oligosaccharides**			
Wild type	1,6-β-Glucotriose	1.7 × 10^6^ ± 1.1 × 10^4^	
Wild type	1,6-β-Glucotetraose	2.6 × 10^6^ ± 3.4 × 10^5^	
Wild type	1,6-β-Glucohexaose	6.0 × 10^6^ ± 6.0 × 10^5^	
Wild type	1,6-β-Glucooctaose	6.4 × 10^6^ ± 1.4 × 10^5^	

*^a^* The kinetic parameter is mg ml^−1^ min^−1^ for pustulan and m^−1^ min^−1^ for glucooligosaccharides.

Based on the presence of a lipoprotein box within its signal peptide, BT3312 is predicted to be located on the outer membrane. Indeed, the ability of Δ*bt3312* to grow on a mixture of 1,6-β-glucooligosaccharides but not pustulan strongly indicates that BT3312 is required to depolymerize the glycan at the bacterial surface, generating molecules of an appropriate size for import through the outer membrane. To provide further support for this hypothesis, whole cell polysaccharide depolymerization assays were performed under aerobic conditions, which prevents transport of oligosaccharides through the outer membrane, and thus report exclusively on the activity of surface enzymes (10, 18). When wild-type *B. thetaiotaomicron*, grown on pustulan or 1,6-β-glucooligosaccharides to activate PUL_1,6-β-glucan_, was subjected to aerobic whole-cell polysaccharide depolymerization assays with pustulan, the bacterium generated a range of glucooligosaccharides, consistent with the activity observed for purified BT3312 (Fig. 1*F*). Equivalent whole-cell assays of the Δ*bt3312* mutant grown on 1,6-β-glucooligosaccharides, which also activate PUL_1,6-β-glucan_, did not produce pustulan-derived oligosaccharides (Fig. 1*F*). Collectively, these data show that BT3312 is located on the surface of *B. thetaiotaomicron*.

##### Crystal structure of BT3312

**Figure 3.**
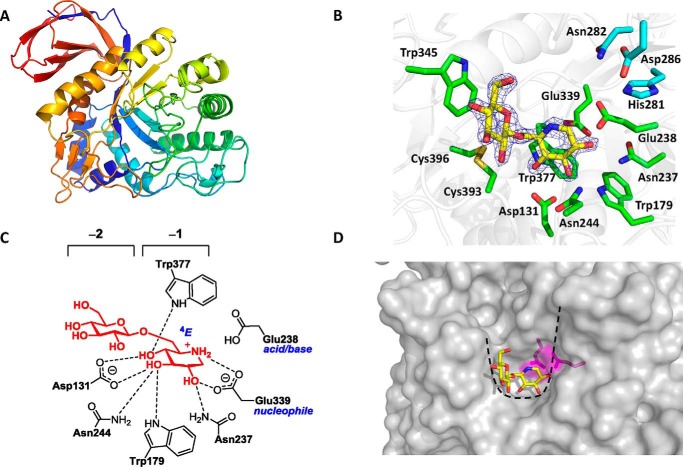
**The structure of BT3312 in complex with GlcDNJ.** *A*, cartoon representation of the structure. *B*, active site residues of BT3312 (*green*) with GlcDNJ (*yellow*) and electron density 2m*F*_o_ − D*F*_c_ map contoured at 1.5 σ (*blue*). *C*, 2D representation of the active site with hydrogen bonds between protein and ligand at −2 and −1 subsites depicted. *D*, surface, colored *gray*, with ligand in *yellow* and catalytic residues Glu^236^ and Glu^337^ in *magenta*. The U-shaped active site is highlighted with a black *dashed line*.

No structure of a GH30_3 enzyme has been reported. Thus, to explore the mechanism of catalysis and substrate binding of an endo-1,6-β-glucanase, the crystal structure of BT3312 was determined in complex with the ligand β-glucosyl-1,6-deoxynojirimycin (GlcDNJ). The structures of the apo- and ligand-bound enzyme were solved by molecular replacement to a resolution of 1.9 and 1.85 Å, respectively, using a GH30 subfamily glucosylceramidase enzyme (PDB code 3RIL) as the search model (Fig. 3 and Table 2). In the ligand complex, unambiguous electron density corresponding to GlcDNJ was evident. The crystal structure revealed that BT3312 comprises two domains. The catalytic domain adopts a (β/α)_8_ barrel fold (TIM barrel), extending from residues Asp^82^ to Lys^427^, and a β-sandwich domain comprising sequences from both the N and C termini (Fig. 3*A*). The TIM barrel contains a central eight-stranded β-barrel, and extending from each β-strand is an α-helix. Extended loops link the α-helices with the β-barrel, and a small β-sheet comprising three anti-parallel strands is inserted into the loop connecting β-strand-8 and α-helix-8. The β-sandwich domain contains seven antiparallel β-strands in one sheet, with two contributed by the N-terminal region of the enzyme. The other β-sheet comprises three antiparallel strands with one derived from the N terminus. Because BT3312 is classified into GH30, it is a member of clan GH-A in which the fold, catalytic apparatus, and mechanism are conserved (19). According to these criteria BT3312 is predicted to use a double-displacement mechanism in which anomeric configuration is retained after bond cleavage. In this two-step mechanism, an enzymic nucleophile performs a nucleophilic attack and generates a glycosyl enzyme intermediate, which in the second step is hydrolyzed. An enzymic residue assists departure of the anomeric group in the first step, acting as a general acid, and assists the hydrolysis of the glycosyl enzyme intermediate in the second step, acting as a general base. The catalytic acid/base and nucleophile have been assigned for a GH30 family member, human β-glucocerebrosidase, and are located at the end of β-strands 4 and 7 of the barrel, respectively (20). Thus, the candidate catalytic residues are Glu^238^ (acid/base) and Glu^339^ (nucleophile). Consistent with this prediction, mutation of either of these residues to Ala resulted in the complete loss of activity (Table 1).

**Table 2:** **Data collection and refinement statistics** The values in parenthesis are for the high resolution shell.

	BT3312 no ligand	BT3312 ligand
**Data collection**		
Date	26/01/14	04/07/15
Source	I02	I03
Wavelength (Å)	0.9794	0.7749
Space group	P2_1_	P2_1_
Cell dimensions		
*a*, *b*, *c* (Å)	62.4, 156.0, 78.0	62.4, 78.8, 145.5
α, β, γ (°)	90.0, 95.0, 90.0	90.0, 100.4, 90.0
No. of measured reflections	429,705 (10,374)	445,926 (22,895)
No. of independent reflections	114,209 (4871)	116,665 (5762)
Resolution (Å)	48.24–1.90 (1.93–1.90)	48.79–1.85 (1.88–1.85)
*CC*_1/2_	0.996 (0.466)	0.996 (0.644)
*I*/σ*I*	9.0 (1.8)	7.8 (1.6)
Completeness (%)	97.4 (97.6)	99.2 (86.2)
Redundancy	3.8 (4.0)	3.8 (2.1)

**Refinement**		
*R*_work_/*R*_free_	18.19/22.12	18.84/22.80
No. atoms		
Protein	10,813	10,738
Ligand/ions	0	68
Water	607	606
B-factors		
Protein	34.3	30.6
Ligand/Ions	N.A.	29.5
Water	37.5	32.5
Root mean square deviations		
Bond lengths (Å)	0.013	0.013
Bond angles (°)	1.53	1.52
PDB code	5NGK	5NGL

**Figure 4.**
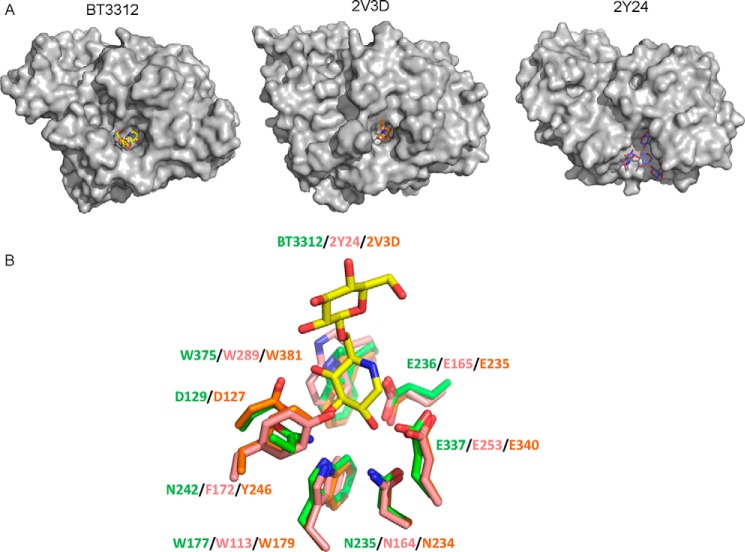
**Comparison of BT3312 with other GH30 enzymes.** *A*, surface representations with ligand of BT3312, *H. sapiens* β-glucosylceramidase (PDB code 2V3D) and the *D. chrysanthemi* glucuronoxylanase (PDB code 2Y24). *B*, comparison of active site conservation between BT3312 (*green*), 2Y24 (*pink*), and 2V3D (*orange*).

Based on analysis of other clan GH-A members, the substrate-binding site of BT3312 is predicted to be positioned on top of the β-barrel. Inspection of this region of the enzyme reveals a deep U-shaped cleft that houses the catalytic residues. The active site cleft of BT3312 adopts a different topology to the substrate-binding regions of other GH-A endo-acting enzymes, which typically display linear or curved clefts that are open at both ends (Figs. 3*D* and 4*A*). Interestingly, NMR analysis of *Candida glabrata* 1,6-β-glucan revealed a hooked, U-shaped conformation (21), which is complementary to the topology of the substrate-binding region of BT3312 (see below).

The structure of BT3312 bound to GlcDNJ reveals details of the active site and the distal substrate-binding subsites (Fig. 3, *B* and *C*). The Glc at the −2 subsite adopts a relaxed chair (^4^*C*_1_) conformation, whereas DNJ bound in the −1 subsite, the active site, is distorted and adopts a ^4^*E* envelope conformation. Substrate distortion at the active site is a characteristic feature of GHs, because distorted conformations require less nuclear movements to achieve the transition state structure and allow the geometric and stereoelectronic requirements of the reaction to be achieved. In particular, GHs undergo reactions involving “exploded” transition states with significant oxocarbonium-ion like character, in which the endocyclic oxygen and anomeric carbon are required to be in the same plane to share the developing positive charge (22). Because most β-gluco-active enzymes proceed through a transition state in a ^4^*H*_3_ (half-chair) conformation and because the ^4^*E* and ^4^*H*_3_ conformations are adjacent to each other on a “Stoddard” conformational plot of pyranose ring conformations, this result is suggestive that this enzyme utilizes a ^1^*S*_3_–^4^*H*_3_^‡^–^4^*C*_1_ conformational itinerary along the reaction coordinate (23, 24). Despite the resemblance of GlcDNJ to the proposed transition state, the inhibitor bound extremely weakly to the enzyme, with an estimated *K_I_* of ∼1 mm, suggesting that additional sugar residues may be required to fully capitalize on interactions along the active site cleft. Indeed, mutagenesis data described below suggest the enzyme has at least four functional subsites for the polysaccharide pustulan. The polar interactions between the active site of BT3312 and GlcDNJ are displayed in Fig. 3 (*B* and *C*). Within the −1 subsite, the carboxylate of Glu^339^ and Nδ2 of Asn^237^ make polar contacts with O_2_. O_3_ interacts with Nϵ1 of Trp^179^, Nδ2 of Asn^244^, and the carboxylate of Asp^131^. The Oδ2 of Asp^131^ and Nϵ1 of Trp^377^ make hydrogen bonds with O_4_. Finally, the endocyclic nitrogen donates a hydrogen bond to Oδ2 of Glu^339^. Binding of GlcDNJ in the active site is also mediated through apolar contacts between the sugar ring and Trp^377^, which provides the hydrophobic platform in the −1 subsite. The catalytic nucleophile, Glu^339^, is 3.2 Å from the anomeric carbon of DNJ and is thus in an appropriate position to mount a nucleophilic attack on the anomeric carbon. Glu^238^ is in an optimal position (3.4 Å) to donate a proton to the glycosidic oxygen appended to C_1_ and thus is able to function as the acid-base catalyst.

The −2 subsite makes only apolar interactions with the Glc in GlcDNJ. Trp^345^ stacks against the pyranose ring, whereas the disulfide formed by Cys^393^ and Cys^396^ also makes apolar contacts with the −2 Glc. Given that O_6_ of the non-reducing glucose points into solvent, it is unlikely that there are additional negative subsites in BT3312. It is difficult to establish the number of positive subsites without bound ligand in this region of the enzyme. However, based on the height of the wall of the substrate-binding cleft, there are likely to be one or possibly two positive sites in which His^281^, Asn^282^, and Asp^286^ contribute to substrate binding. To examine the contribution of these residues to catalysis by BT3312, alanine-scanning mutagenesis was performed (Table 1). As stated above, the catalytic residues including Glu^238^ are essential for activity. Mutation of the other residues in the active site caused a significant reduction in the activity observed, except Asp^286^, which only resulted in a 3-fold loss of catalytic efficiency. The relatively few polar interactions, which are confined to the active site, suggest that specificity is driven by the complementary conformation of the substrate and the topology of the enzyme. The biochemical data presented above point to only one positive subsite, which is surprising because the crystal structure of the enzyme suggests four observable subsites extending from −2 to +2. It is possible that the U-shaped structure of the substrate, which is required to fully occupy the four subsites, can only be achieved through extensive intrachain hydrogen bonds within the polysaccharide. Thus, short oligosaccharides may not be able to adopt the U-shaped conformation required to occupy the +2 subsite.

Comparison of the structure of BT3312 with GH30 enzymes with different specificities sheds light on the structural basis for substrate recognition in this family. These structural comparisons show that specificity is dominated by the topology of the substrate-binding regions of the respective proteins (Fig. 4, *A* and *B*). Thus, when BT3312 is compared with the glucuronoxylanase from *Dickeya chrysanthemi* D1 (PDB code 2Y24), the *B. thetaiotaomicron* enzyme contains a long loop extending from residues 381–398 that sterically occludes the −2 and −3 subsites in the glucuronoxylanase (25). This loop is stabilized by a disulfide bond between Cys^393^ and Cys^396^, and three short β strands behind the loop. BT3312 contains a second extended loop comprising Gln^236^ to Trp^252^ that prevents access to the positive subsites in the glucuronoxylanase. The lack of residues that target O_6_ of the substrate bound at the −1 or −2 subsites reinforces the view that it is the topology of the proximal substrate region of BT3312 that confers specificity for 1,6-β-glucan over 1,4-β-xylan. 1,4-β-Xylan, which lacks O_6_ substitution, adopts a linear 3-fold screw axis conformation (26, 27) that is distinct from the highly curved, U-shaped structure exhibited by 1,6-β-glucan (21).

The active site (−1) subsite of BT3312 was compared with the *Homo sapiens* β-glucosylceramidase (PDB code 2V3D) and the *D. chrysanthemi* glucuronoxylanase (PDB code 2Y24), representatives of subfamilies GH30_1 and GH30_8, respectively. In addition to the catalytic nucleophile and acid-base residues, many of the other substrate-binding amino acids are conserved in the three enzymes. Indeed the only two residues in the active site of BT3312 that are not conserved in other GH30 enzymes are Asn^244^ and Asp^131^. The asparagine in BT3312 is replaced in the other two enzymes by an aromatic residue that makes apolar contacts with the substrate. Though Asp^131^ is conserved in the β-glucosylceramidase, the loop containing this residue adopts a different position in the glucuronoxylanase (to accommodate the linear extended substrate), and thus there is no residue equivalent to the aspartate in this enzyme. The additional distal subsites in the glucuronoxylanase likely compensate for this loss of binding energy in the active site that would have been provided by this aspartate.

##### Surface glycan-binding proteins

**Figure 5.**
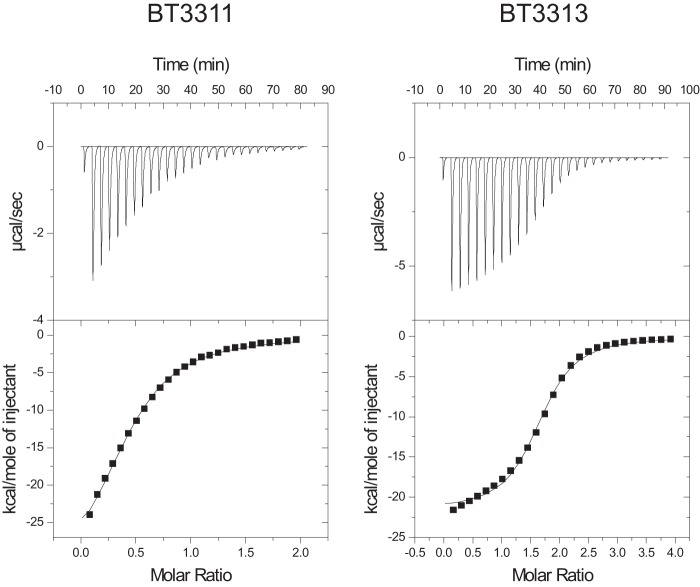
**Isothermal titration calorimetry of glycan-binding proteins.** Shown are representative traces of the binding of 50 μm SusD BT3311 (*left panel*) or SGBP BT3313 (*right panel*) to 5 mg ml^−1^ 1,6-β-glucan in 50 mm HEPES, pH 7.5 (estimated at 1 mm to fit the data).

Similar to other characterized PULs, PUL_1,6-β-glucan_ encodes two putative surface lipoproteins predicted to contribute to polysaccharide binding. The genes encoding both BT3311, the SusD-like protein, and BT3313, a predicted lipoprotein, were expressed in *Escherichia coli*, and their ability to bind glucans was evaluated. Affinity gel electrophoresis showed that both BT3311 and BT3313 bound specifically to 1,6-β-glucan and showed no affinity for laminarin (1,3-β-glucan) or hydroxy-ethyl cellulose (1,4-β-glucan) (data not shown). These data demonstrate that BT3313 is an SGBP and, as with the SusD-like protein BT3311, specifically target the glycan depolymerized by the enzymes encoded by PUL_1,6-β-glucan_. Isothermal titration calorimetry was used to quantify the thermodynamics of binding to polysaccharide and 1,6-β-glucooligosaccharides generated from limited digestion of 1,6-β-glucan with BT3312 (Fig. 4). The data are presented in Table 3, and example binding curves are shown in Fig. 5. The SusD-like protein bound to 1,6-β-glucan with a *K_a_* value of 4 × 10^4^ m^−1^ and with similar affinity to glucoheptaose and glucohexaose. Affinity for glucopentaose was an order of magnitude lower, showing that the SusD-like protein most likely has six sugar binding subsites. BT3313 showed ∼10-fold higher affinity for 1,6-β-glucan than BT3311. Similar dependence on size of oligosaccharide is seen with a preference for oligosaccharides with a degree of polymerization ≥ 6, although the largest oligosaccharide tested, glucoheptaose, did not bind as tightly as the polysaccharide. As proposed for the enzyme BT3312, it may be that significantly longer oligosaccharides than heptaose are necessary to form the U-shaped conformation required for maximal binding, although the possibility that the protein contains >7 sugar binding sites cannot be discounted.

**Table 3:** **ITC binding data for BT3311 and BT3313**

Protein	Ligand	*K_a_*	Δ*H*	*N*
		*m*^−*1*^	*kcal*	
BT3311	1,6-β-Glucan (fit as 1 mm)	6.5 × 10^4^ ± 0.2	−19 × 10^4^ ± 0	0.8 ± 0.02
BT3311	1,6-β-Glucoheptaose	2.1 × 10^4^ ± 0.1	−14 × 10^4^ ± 0	1.2 ± 0.02
BT3311	1,6-β-Glucohexaose	1.6 × 10^4^ ± 0.1	−14 × 10^4^ ± 1	1.0 ± 0.04
BT3311	1,6-β-Glucopentaose	8.3 × 10^3^ ± 0.3	−16 × 10^4^ ± 3	1.1 ± 0.1
BT3311	1,6-β-Glucotetraose	Low*^a^*	Low	Low
BT3311	1,6-β-Glucobiose	NB*^b^*	NB	NB
BT3313	1,6-β-Glucan (fit as 1 mm)	5.3 × 10^5^ ± 0.1	−36 × 10^4^ ± 1	1.02 ± 0.04
BT3313	1,6-β-Glucoheptaose	1.5 × 10^4^ ± 0.2	−24 × 10^4^ ± 3	1.23 ± 0.1
BT3313	1,6-β-Glucohexaose	8.0 × 10^3^ ± 0.7	−15 × 10^4^ ± 2	1.38 ± 0.2
BT3313	1,6-β-Glucopentaose	3.6 × 10^3^ ± 0.2	−15 × 10^4^ ± 3	1.28 ± 0.2
BT3313	1,6-β-Glucotetraose	Low	Low	Low
BT3313	1,6-β-Glucobiose	NB	NB	NB

*^a^* Low, binding too low to quantify.

*^b^* NB, no binding was evident.

### Periplasmic processing of 1,6-β-glucooligosaccharides

Once glycans are bound and cleaved at the cell surface, oligosaccharides are typically imported by the SusCD-like TonB-dependent transporter complex. The GH3 enzyme BT3314 contains a type I signal peptide and is predicted to be located in the periplasm where it can depolymerize imported 1,6-β-glucooligosaccharides into glucose, which is then metabolized within the cytoplasm. Family GH3 contains a range of exo-acting GHs including β-glucosidases (28). BT3314 is a β-glucosidase that can cleave 1,3- and 1,4-β-glucobiose but displays ∼30-fold higher activity for the 1,6-β-linkage (Table 4). The *k*_cat_/*K_m_* for 1,6-β-glucotriose and glucobiose were similar, demonstrating that the enzyme likely has only two subsites. Although the catalytic efficiency of BT3314, *k*_cat_/*K_m_* = 6 × 10^6^ m^−1^ min^−1^, is in the range observed for other β-glucosidases, *bt3314* transcription in response to pustulan was 10-fold lower than the other genes in PUL_1,6-β-glucan_. It is possible that the slow degradation of imported 1,6-β-glucooligosaccharides allows persistence of higher concentrations of the “inducing ligand” of PUL_1,6-β-glucan_, enabling the locus to remain up-regulated during growth on pustulan.

**Table 4:** **Kinetic parameters of BT3314 and BACOVA_00946**

Enzyme	Substrate	*K_m_*	*k*_cat_	*k*_cat_/*K_m_*
		μ*m*	*min*^−*1*^	μ*m*^−*1*^ *min*^−*1*^
BT3314	pNP-β-d-Glc*^a^*	2403 ± 379	40.82 ± 2.9	0.02
BT3314	1,3-β-Glucobiose	ND*^b^*	ND	0.18 ± 0.01
BT3314	1,4-β-Glucobiose	ND	ND	0.05 ± 0.01
BT3314	1,6-β-Glucobiose	ND	ND	5.6 ± 0.2
BT3314	1,6-β-Glucotriose	ND	ND	6.7 ± 0.6
BACOVA_00946	pNP-β-d-Glc	1052 ± 87	195.8 ± 5.5	0.19
BACOVA_00946	1,3-β-Glucobiose	ND	ND	3.73 ± 0.1
BACOVA_00946	1,4-β-Glucobiose	ND	ND	0.65 ± 0.02
BACOVA_00946	1,6-β-Glucobiose	ND	ND	38.8 ± 0.8

*^a^* pNP-β-d-Glc, 4-nitrophenyl β-d-glucopyranoside.

*^b^* ND, not determined.

### Distribution of the PUL_1,6-β-glucan_ within the Bacteroidetes

**Figure 6.**
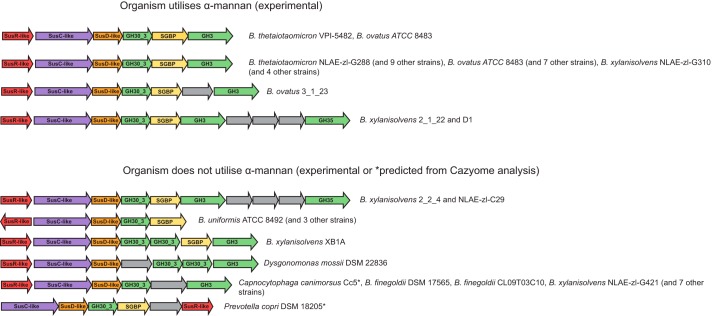
**Distribution of PUL_1,6-β-glucan_ in Bacteroidetes.** PULs with synteny to PUL_1,6-β-glucan_ were identified using the Integrated Microbial Genome and Microbiome sample database at www.img.jgi.doe.gov using the Gene Ortholog Neighborhood viewer and PULDB at www.cazy.org/PULDB.^5^ Example PULs from strains that can and cannot grow on α-mannan are labeled. For those strains that have not been tested for growth on α-mannan, www.cazy.org was searched for genes encoding GH76 α-mannanases and GH38 α-mannosidases; those strains lacking the genes were considered as unlikely to grow on α-mannan are indicated by *asterisks*. The functions or enzyme families of the gene products are indicated. The basis for assigning genes encoding SGBPs was based on significant sequence identity (>35%) with BT3313 (SGBP of *B. thetaiotaomicron*) and the presence of a lipoprotein signal peptide. The *gray* genes encode proteins of unknown function.

The distribution of the PUL_1,6-β-glucan_ within other Bacteroidetes genomes was examined using the Gene Ortholog Neighborhood viewer on the Integrated Microbial Genome and Microbiome sample database at img.jgi.doe.gov, and the Polysaccharide Utilization Loci database (PULDB; www.cazy.org/PULDB) (29).^5^ Manual comparison of the strains containing a PUL with synteny to PUL_1,6-β-glucan_ to the ∼350 strains screened for growth on α-mannan in Ref. 10 revealed that the PUL is more widely distributed than the ability to degrade α-mannan, which is mostly limited to strains of *B. thetaiotaomicron*, *Bacteroides ovatus*, and *Bacteroides xylanisolvens* (Fig. 6). Although several of the mannan-degrading strains possess a putative PUL_1,6-β-glucan_, the Bacteroidetes *Capnocytophaga canimorsus* (a commensal of the canine mouth and occasional human pathogen) and *Prevotella copri*, which are not predicted to grow on yeast α-mannan because of a lack of α-mannanases and mannosidases encoded in the genome, also contain a PUL that displays substantial synteny with PUL_1,6-β-glucan_. The functional significance of syntenic PULs to PUL_1,6-β-glucan_ is illustrated by the observation that (i) *B. ovatus* can grow on pustulan, (ii) pustulan up-regulates the locus, and (iii) *bacova_00946* encodes a β-glucosidase that displays a strong preference for 1,6-β-glucooligosaccharides (Fig. 1, *A* and *C*, and Table 4).

### Conclusions

**Figure 7.**
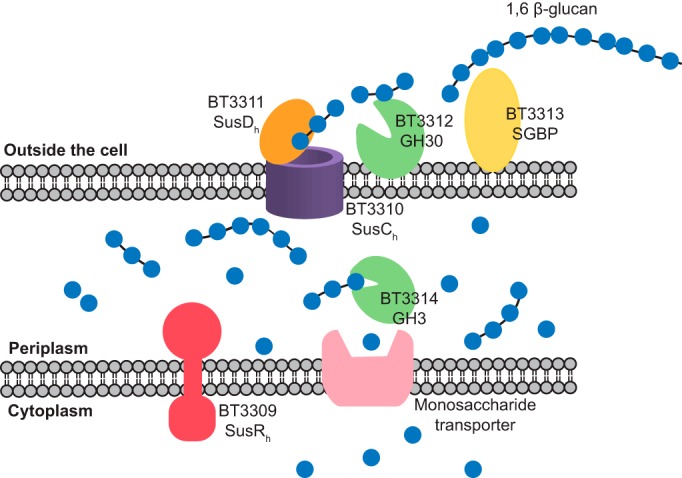
**Cartoon representation of 1,6-β-glucan degradation by *B. thetaiotaomicron*.** A schematic of 1,6-β-glucan degradation by *B. thetaiotaomicron* is shown. Gene products are colored as in Fig. 1*A*. 1,6-β-Glucan is represented by *blue circles*. The inner membrane transporter to transport monosaccharides into the cytoplasm for fermentation is assumed to exist but is not within PUL_1,6-β-glucan_.

Our data show that PUL_1,6-β-glucan_ (*bt3309–bt3314*) does not depolymerize mixed linkage 1,3;1,4-β-glucans from cereals, as proposed previously for *B. ovatus* (1); rather, it is highly specific for fungal 1,6-β-glucans. A model for the degradative pathway of the glucan is shown in Fig. 7. The critical enzyme encoded by the PUL is BT3312, an endo-1,6-β-glucanase that is displayed on the bacterial surface, allowing direct access to the intact glycan. The SusD-like protein and SGBP show a preference for longer substrates, suggesting that the secondary structure of the polysaccharide is required for recognition by these proteins. The “hook-like” secondary structure of 1,6-β-glucan is distinct from other β-glucans, and the U-shaped topology of the substrate-binding cleft of the endo-1,6-β-glucanase, which makes limited polar interactions with the ligand GlcDNJ, matches the conformation of its target glucan, suggesting that it is shape complementarity between substrate and enzyme that is the driving force for substrate specificity. It has been shown that a GH30_3 enzyme from *Aspergillus fumigatus* promotes the release of cell wall proteins from *C. albicans* cell walls, which suggests that another role for PUL_1,6-β-glucan_ could be to increase access of yeast cell wall components for other *B. thetaiotaomicron* enzyme systems, such as the α-mannan-degrading PUL (30). 1,6-β-glucan is also a common component of fungal cell walls in mushrooms, as well as gut commensal fungi such as *S. cerevisiae*, *C. glabrata*, and *C. albicans* (the latter pair are also important human pathogens). The observation that this PUL is more widely distributed within the *Bacteroidetes* than the α-mannan PULs could reflect the multiple sources of this glycan from both mannan-free and mannan-rich fungi and yeasts. The presence of the locus in bacteria that do not use the more abundant mannan glycans in *S. cerevisiae* suggests that its primary role in those organisms may be to target 1,6-β-glucan from edible mushrooms.

## Materials and methods

### Bacterial strains

*B. thetaiotaomicron* VPI-5482 and *B. ovatus* ATCC 8483 were cultured anaerobically in TYG medium, brain-heart infusion (Sigma) plus 2% (w/v) agar or minimal medium containing 0.5% (w/v) 1,6-β-glucan (Elicityl Oligotech) or glucose as sole carbon source, as described previously (2).

### Gene expression studies

Comparison of the levels of transcript expression from the PUL was performed by RT-PCR. *B. thetaiotaomicron* or *B. ovatus* were cultured in 5 ml of minimal medium containing 0.5% (w/v) carbon source, as described above. Triplicate bacterial cultures were harvested at mid-log phase (*A*_600_ ∼0.8), placed in RNAprotect (Qiagen), and then stored at −80 °C overnight, before purification with RNeasy kit (Qiagen). RNA purity was assessed spectrophotometrically, and 1 μg of RNA was used immediately for reverse transcription (QuantiTect reverse transcription kit; Qiagen). RT-PCR was performed in a 96-well plate on a LightCycler 96 System (Roche) with FastStart Essential DNA Green Master (Roche) using the primers shown in supplemental Table S1. The reactions were carried out in 10 μl, consisting of 5 μl of SYBR Green mix, 20 ng of cDNA, and 1 μm (PUL genes) or 0.125 μm (16 S rRNA) primer mix. The reaction conditions were 95 °C 600 s, followed by 45 cycles of 95 °C for 10 s, 55 °C for 10 s, and 72 °C for 10 s. *C*_q_ values were calculated using LightCycler 96 SW 1.1. The data were normalized to 16 S rRNA transcript levels, and changes in expression level were calculated as fold change compared with cultures of minimal medium plus glucose.

### Oligosaccharide production

One gram of 1,6-β-glucan (Elicityl Oligotech) was digested with 50 nm BT3312 in 20 mm Tris, pH 8.0, for 30 min to produce small oligosaccharides or by acid hydrolysis with 20 mm HCl for 2 h to generate longer oligosaccharides. The mixture was subjected to size-exclusion chromatography on two Biogel P2 columns (120 × 2.5 cm) in series in 50 mm acetic acid. Fractions were screened by thin layer chromatography, and those containing pure oligosaccharide were pooled and freeze-dried.

### Recombinant protein production

Genes encoding proteins of interest from PUL_1,6-β-glucan_ were amplified by PCR from *B. thetaiotaomicron* VPI-5482 genomic DNA and cloned into a pET28 derivative, including an N-terminal hexahistidine tag, with any native signal peptide removed. Proteins were expressed in *E. coli* Tuner cells by culturing cells in Luria broth with 10 mg ml^−1^ kanamycin to *A*_600_ of ∼1.0 at 37 °C before cooling to 16 °C and inducing with 1 mm isopropyl β-d-1-thiogalactopyranoside overnight. The cells were harvested by centrifugation at 5000 × *g* for 5 min and resuspended in 20 mm Tris-HCl buffer, pH 8.0, containing 300 mm NaCl (buffer A). The cells were lysed by sonication, and the cell-free extract was recovered by centrifugation at 15,000 × *g* for 30 min. The proteins were purified from the cell-free extract using immobilized metal affinity chromatography using Talon^TM^, a cobalt-based matrix. Proteins were eluted from the column in buffer A containing 100 mm imidazole. For crystallization trials, immobilized metal affinity chromatography-purified protein was concentrated and further purified by gel filtration chromatography using a Superdex S200 16/600 column equilibrated in buffer A. Protein concentration was measured at 280 nm using a NanoDrop spectrophotometer.

### Enzyme assays

Reducing sugar assays on polysaccharide were carried out in buffer A, including 0.1 mg ml^−1^ BSA, following the method of Miller (31). Briefly, 2 mg ml^−1^ 1,6-β-glucan (Pustulan, Elicityl Oligotech; containing ∼2% *O-*acetylation) was incubated with concentrations of enzyme from 50 nm to 1 mm at 37 °C. A FLUOstar Omega microplate reader (BMG Labtech) was used to measure activity in 96-well plates. Substrate depletion assays against oligosaccharides were performed in 50 mm sodium phosphate, 100 mm sodium chloride, pH 7.0 buffer. Enzyme (1–10 nm) was incubated with oligosaccharide (100 μm), and aliquots were taken at intervals over 60 min. The samples were subjected to high-pressure anion-exchange chromatography (HPAEC) using an ICS-3000 machine with a Carbopac PA 200 column. The oligosaccharides were eluted from the column using a 20 or 60% gradient of 100 mm sodium hydroxide and 1 m sodium acetate. The peak areas of oligosaccharides were monitored over time and plotted in GraphPad Prism to give the *k*_cat_/*K_m_* of the enzyme for each oligosaccharide. Exo-acting enzyme activity was measured by monitoring PNP release at 400 nm or the production of glucose over time at 340 nm using the d-glucose/d-mannose/d-fructose kit (Megazyme), as per the manufacturer's instructions. Initial rates were measured, and kinetic parameters were determined from this using GraphPad Prism. *K_I_* for GlcDNJ against BT3312 was estimated by assaying glucose production from glucotriose using the d-glucose detection kit in the presence of GlcDNJ (0–1.5 mm).

### Whole cell assays

*B. thetaiotaomicron* WT or Δ*bt3312* cells from three replicate wells of a 96-well plate, cultured on minimal medium plus glucose or digested 1,6-β-glucan were pooled (600 μl total) and washed twice in 1 ml 50 mm sodium phosphate, 100 mm sodium chloride, pH 7.0. Washed cells were resuspended in a total volume of 200 μl of the same buffer, containing 2 mg ml^−1^ 1,6-β-glucan and incubated at 37 °C overnight. To analyze the reactions, the cells were pelleted by centrifugation at 13,000 × *g* for 1 min, and the supernatant was subjected to HPAEC as described above.

### Isothermal titration calorimetry

Titrations were carried out in a Microcal VP isothermal titration calorimetry machine, in 50 mm Na-Hepes buffer, pH 7.5, at 25 °C. The reaction cell contained protein at 50 μm, and the syringe contained either the oligosaccharides (2–5 mm) or polysaccharide (4–5 mg ml^−1^). The titrations were analyzed using Microcal Origin version 7.0 software to derive *K_a_* values.

### Crystallography and structure determination

Native BT3312 was crystallized at 20 mg ml^−1^ in 0.2 m sodium bromide, 0.1 m Bis-Tris propane, pH 6.5, 20% w/v PEG 3350. For ligand bound structure, BT3312 was crystallized in 0.2 m potassium thiocyanate 20% w/v PEG 3350 and soaked in 8.6 mm GlcDNJ. Crystallization sitting drops were 100 or 200 nl of protein sample supplemented with 100 nl of reservoir using a Mosquito nanolitre pipetting robot (TTP Labtech). All samples were cryo-protected using 20% PEG 400. The phase problem was solved by molecular replacement for the apo data set with 3RIL as search model using MrBump (32). The ligand-containing data set was solved using the apo model as search model using MolRep (33). The initial model was improved using the automated model building program Arp_warp (34). Recursive cycles of model building in COOT (35) and refinement in Refmac5 (36) were performed to produce the final model. Solvent molecules, ligands, and ions were added using COOT and checked manually. All other computing used the CCP4 suite of programs (37). Five percent of the observations were randomly selected for the *R*_free_ set. The models were validated using MolProbity (38). The data statistics and refinement details are reported in Table 2.

### Synthesis of β-glucosyl-1,6-deoxynojirimycin (GlcDNJ)

#### 

##### 2,3,4-Tri-O-benzoyl-N-benzyloxycarbonyl-deoxynojirimycin (2)

TBDMSCl (24 mg, 0.16 mmol) was added to a mixture of **1** (39) (40 mg, 0.13 mmol) and imidazole (22 mg, 0.33 mmol) in dry dimethylformamide (1.0 ml) at 0 °C. After 2 h, TLC (CHCl_3_:MeOH, 19:1) showed conversion to a non-polar compound. The mixture was cooled to 4 °C, and then pyridine (1.0 ml), 4-dimethylaminopyridine (1.3 mg, 0.012 mmol), and benzoic anhydride (176 mg, 0.77 mmol) were added. The solution was stirred overnight at room temperature and then quenched with MeOH (1 ml). The mixture was diluted with EtOAc (15 ml) and then washed with brine (2 × 10 ml), saturated NaHCO_3_ (10 ml), and water (10 ml). The organic phase was dried (MgSO_4_), filtered, and concentrated. The residue was purified by flash chromatography (AcOEt:hexane, 19:1 to 6:4), yielding the silylated ester as a white foam. [α]_D_^25 −^3.2 (*c* 0.68, CHCl_3_); ^1^H NMR (500 MHz, d_6_-DMSO, 65 °C): δ −0.04 (3 H, s, Me), 0.02 (3 H, s, Me), 0.81 (9 H, s, C-Me), 3.77 (1 H, d, *J*_1,1a_ = 15.4 Hz, H1), 3.97 (1 H, dd, *J*_6,6a_ = 10.5, *J*_5,6_ = 7.7 Hz, H6), 4.08 (1 H, dd, *J*_6,6a_ = 10.5, *J*_5,6a_ = 7.4 Hz, H6a), 4.45 (1 H, d, *J*_1,1a_ = 15.4 Hz, H1a), 4.69 (1 H, app. t, *J*_4,5_ = *J*_5,6_ = *J*_5,6a_ = 7.2 Hz, H5), 5.07 (1 H, d, *J* = 12.6 Hz, CH_2_Ph), 5.02 (1 H, d, *J* = 12.6 Hz, CH_2_Ph), 5.24 (1 H, d, *J*_1,2_ = 2.1, H2), 5.47 (1 H, m, H4), 5.50 (1 H, t, *J*_2,3_ = 3.0 Hz, H3), 7.14–8.09 (20 H, m, Ar); ^13^C-NMR (101 MHz, d_6_-DMSO, 25 °C): δ −2.4, −2.3 (2 × 1C, Me), 17.7 (3 C, CMe), 25.5 (1 C, CMe_3_), 38.8 (1 C, C1), 55.6 (1 C, C5), 59.2 (1 C, C6), 66.5 (1 C, C4), 66.6 (1 C, CH_2_Ph), 67.2 (1 C, C3), 67.7 (1 C, C2), 126.0, 127.1, 127.7, 128.2, 128.5, 128.6, 128.7, 128.8, 128.9, 129.1, 129.28, 129.30, 129.8, 133.6, 134.0, 136.4 (24 C, Ar), 155.6, 163.9, 164.5, 164.8 (4 × C, C = O); HRMS (ESI^+^) *m*/*z* 724.2941 (C_41_H_46_NO_9_Si (M + H)^+^ requires 724.2936).

The silylated ester (68 mg, 0.094 mmol) was dissolved in AcOH:H_2_O:THF (3:1:1, 9.0 ml) and the mixture was stirred overnight at 40 °C. The solution was concentrated under reduced pressure, and the residue was purified by flash chromatography (AcOEt/hexane, 19:1 to 6:4) to afford the alcohol **2** (55 mg, 70% over three steps) as a colorless oil, [α]_D_^25 −^3.3 (*c* 1.25, CHCl_3_); ^1^H NMR (500 MHz, d_6_-DMSO, 65 °C): δ 3.74 (1 H, d, *J*_1,1a_ = 15.6 Hz, H1), 3.81–3.93 (2 H, m, H6 and H6a), 4.43 (1 H, d, *J*_1,1a_ = 15.6 Hz, H1a), 4.69 (1 H, app. t, *J*_4,5_ = *J*_5,6_ = *J*_5,6a_ = 7.5 Hz, H5), 5.00, (1 H, t, *J*_OH,6_ = *J*_OH,6a_ = 6.0 Hz, OH), 5.00 (1 H, d, *J* = 12.7 Hz, CH_2_Ph), 5.08 (1 H, d, *J* = 12.7 Hz, CH_2_Ph), 5.20 (1 H, d, *J*_1,2_ = 2.0, H2), 5.47 (1 H, m, H4), 5.50 (1 H, t, *J*_2,3_ = 3.0 Hz, H3), 7.10–8.09 (20 H, m, Ar); ^13^C-NMR (101 MHz, d_6_-DMSO, 25 °C): 39.1 (1 C, C1), 56.0 (1 C, C5), 57.7 (1 C, C6), 66.7 (1 C, CH_2_Ph), 66.5, 67.2, 67.7 (3 × C, C2,3,4), 125.3, 127.2, 127.7, 128.2, 128.6, 128.9, 129.3, 129.6, 133.6, 133.9, 136.4 (24 C, Ar), 155.8, 164.0, 164.6, 164.7 (4 × 1C, C = O); HRMS (ESI^+^) *m*/*z* 610.2033 (C_35_H_32_NO_9_ (M + H)^+^ requires 610.2072).

##### 6-O-(2-O-acetyl-3,4,6-tri-O-benzyl-d-β-glucopyranosyl)-2,3,4-tri-O-benzoyl-N-benzyloxycarbonyl-deoxynojirimycin (4)

A mixture of 2-*O*-acetyl-3,4,6-tri-*O*-benzyl-d-glucopyranosyl trichloroacetimidate **3** (40) (20 mg, 0.027 mmol), acceptor **2** (13.0 mg, 0.021 mmol) and freshly activated 4 Å molecular sieves in dry toluene (1.5 ml) was stirred at −30 °C under N_2_ for 1 h. TfOH in toluene (1% v/v, 8 μl, 0.0006 mmol) was then added, and the mixture allowed to warm to −10 °C and stirred for 30 min. The reaction was quenched with triethylamine (0.1 ml). The mixture was filtered through Celite and rinsed with toluene, and the solvent was evaporated under reduced pressure. The residue was purified by flash chromatography (19:1 to 6:4 hexane/EtOAc) to afford **4** as a colorless glass (20 mg, 87%). [α]_D_^25^ +1.0 (*c* 0.8, CHCl_3_); ^1^H NMR (500 MHz, d_6_-DMSO, 65 °C): δ 1.95 (3 H, s, Me), 3.60 (2 H, m, H3′ and H5′), 3.66 (2 H, m, H6′ and H6a′), 3.76 (2 H, m, H1 and H4′), 4.02 (1 H, dd, *J*_6,6a_ = 10.3, *J*_5,6_ = 8.9 Hz, H6), 4.13 (1 H, dd, *J*_6,6a_ = 10.7, *J*_5,6a_ = 5.3 Hz, H6a), 4.42 (1H, d, *J*_1,1a_ = 15.7 Hz, H1a), 4.45 (1 H, d, *J* = 12.2 Hz, CH_2_Ph), 4.50 (1 H, d, *J* = 12.2 Hz, CH_2_Ph), 4.57 (1 H, d, *J* = 11.4 Hz, CH_2_Ph), 4.61 (1 H, d, *J* = 11.4 Hz, CH_2_Ph), 4.68 (1 H, d, *J*_1′,2′_ = 8.0 Hz, H1′), 4.73 (2 H, d, *J* = 11.9 Hz, CH_2_Ph), 4.77 (1 H, d, *J*_1′,2′_ = 8.0 Hz, H2′), 4.83 (1 H, m, H5), 4.97 (1 H, d, *J* = 12.6 Hz, CH_2_Ph), 5.06 (1 H, d, *J* = 12.6 Hz, CH_2_Ph), 5.18 (1H, d, *J*_2,3_ = 1.4 Hz, H2), 5.29 (1 H, m, H4), 5.52 (1 H, m, H3), 7.04–8.09 (35 H, m, Ar); ^13^C NMR (101 MHz, d_6_-DMSO): δ 20.5 (1 C, Me), 53.8 (1 C, C5), 64.6 (1 C, C6), 66.8 (1 C, CH_2_Ph), 67.3 (1 C, C4), 67.3 (1 C, C3), 67.8 (1 C, C2), 68.4 (1 C, C6′), 72.3, 72.5, 74.06, 74.13 (5 C, 3 × CH_2_Ph, C2′ and C5′), 77.8 (1 C, C3′), 81.8 (1 C, C4′), 100.0 (1 C, C1′), 127.2, 127.4, 127.6, 127.7, 127.9, 128.20, 128.25, 128.6, 128.9, 129.3, 129.6, 133.6, 133.7, 133.9, 138.0, 138.1, 138.3 (42 C, Ar), 155.6, 164.0, 164.6, 164.7, 169.1 (5 × 1 C, C = O); HRMS (ESI^+^) *m*/*z* 1101.4268 (C_64_H_65_N_2_O_15_ (M + NH_4_)^+^ requires 1101.4379).

##### β-d*-Glucopyranosyl-1,6-deoxynojirimycin (GlcDNJ, 5)*

Sodium methoxide solution (0.10 m, 1.0 ml, 0.10 mmol) was added to a solution of **4** (13 mg, 0.012 mmol) in CH_2_Cl_2_/MeOH (1:1, 2 ml), and the solution was stirred at room temperature. for 1 h. The mixture was neutralized with Dowex-50 resin (H^+^ form), filtered, and concentrated under reduced atmosphere overnight. The reaction washed thoroughly with MeOH:H_2_O (2:1), and concentrated under reduced pressure. The residue was purified by ion-exchange chromatography (Dowex 1X-8, OH^−^ form, eluted with H_2_O; Amberlite CG50 Type 1, H^+^ form, eluted with H_2_O, and then 6 m aqueous NH_3_) followed by C_18_ reversed phase chromatography (CH_3_CN:H_2_O, 95:5), to afford the azasugar **5** (2.9 mg, 75%) with NMR data matching the previously reported (41). [α]_D_^23^ +7.7 (c 0.1, H_2_O); HRMS (ESI^+^) *m*/*z* 326.1460 (C_12_H_24_NO_9_ (M + H)^+^ requires 326.1446) (41).

### Genetic manipulation of B. thetaiotaomicron

The gene *bt3312* gene was removed from the genome of *B. thetaiotaomicron* by in-frame deletion using the vector pExchange, as described in Ref. 42.

## Author contributions

G. S. and S. J. W. carried out the chemical synthesis. F. C. and E. C. L. performed the microbiology and microbial genetics. The biochemistry was carried out by F. C., E. C. L., M. J. T., and N. H. A. B. collected, solved, and deposited crystallographic data. The paper was written primarily by E. C. L. with some input from H. J. G. and S. J. W. Experimental design and data interpretation was by E. C. L., F. C., H. J. G., and S. J. W.

## Supplementary Material

Supplemental Data

## Footnotes

5 Please note that the JBC is not responsible for the long-term archiving and maintenance of this site or any other third party hosted site.

## Footnotes

4 The abbreviations used are:

PUL: polysaccharide utilization locus
PDB: Protein Data Bank
SGBP: surface glycan-binding protein
GH: glycoside hydrolase
TYG: tryptone-yeast extract-glucose
GlcDNJ: β-glucosyl-1,6-deoxynojirimycin
HPAEC: high-pressure anion-exchange chromatography.
