# Supplementary material for: A Bacteroidetes locus dedicated to fungal 1,6-β-glucan degradation: Unique substrate conformation drives specificity of the key endo-1,6-β-glucanase
Source: J Biol Chem. 2017 May 1;292(25):10639–50. doi: 10.1074/jbc.M117.787606 (PMC5481569; doi:10.1074/jbc.M117.787606)
Supplement: Supplemental Data [file supp_292_25_10639__index.html]

A Bacteroidetes locus dedicated to fungal 1,6-β-glucan degradation: unique substrate conformation drives specificity of the key endo-1,6-β-glucanase — A Bacteroidetes locus dedicated to fungal 1,6-β-glucan degradation: Unique substrate conformation drives specificity of the key endo-1,6-β-glucanase — Bacterial metabolism of 1,6-β-glucan — Supplemental Data 

# A Bacteroidetes locus dedicated to fungal 1,6-β-glucan degradation: Unique substrate conformation drives specificity of the key endo-1,6-β-glucanase

## Supplemental Data

- Supplemental Data (.pdf, 148 KB) - Primers used in this study
