# Supplementary material for: A Bacteroidetes locus dedicated to fungal 1,6-β-glucan degradation: Unique substrate conformation drives specificity of the key endo-1,6-β-glucanase
Source: J Biol Chem. 2017 May 1;292(25):10639–50. doi: 10.1074/jbc.M117.787606 (PMC5481569; doi:10.1074/jbc.M117.787606)
Supplement: Supplemental Data [file 10.1074_M117.787606_jbc.M117.787606-1.pdf]

Table S1 Primers for RT-PCR

Bacterium	Open reading frame	Sequence (5'- 3')
B. thetaiotaomicron	*bt3309*	TGCCTCTTCGGCTTCTATTT
		ATGGAATTAGGAGCCACGTC
	bt3310	ACGTGTTGCTTACCTTGCTG
		GTCGGAGGCTTCACCATACT
	bt3311	TCCGTAGCCCGTACTCTTCT
		AAACCATCAGCCTTCACCTC
	bt3312	ATGGAAGAGGTAGCATTGGG
		TGACAACCACCTTCACGATT
	bt3313	GGCTGGGAAGACAATACGAT
		CGGACTCGCTTCATAGTTGA
	bt3314	GAGTTCAACCGTCACACCAC
		ACCGGAGTCGGTATCTTCAC
B. ovatus	*bacova_00941*	TATCTGCGACTCTGCAATCC
		CGTGTACATTCCCGTTGAAG
	bacova_00942	GGTAACCAGTCGGGTATTGG
		AGCATTTGCGTAATCAGTCG
	bacova_00943	GGACTGGAACCGGTATATGG
		CATGGCACGGAATATCTTTG
	bacova_00944	GGCGCCTACTTCCATTACAT
		TCAGAGAACGTTTCCGTCAG
	bacova_00945	ATCTGGGCTATTGGAACAGG
		CCGGCTATGAATGTCAGTTG
	bacova_00946	AGTGGCTAAAGATGCCGACT
		TGCCAGTTCGCTTATCACTC
